# Silencing sounds off

**DOI:** 10.7554/eLife.06717

**Published:** 2015-03-02

**Authors:** Yu-Fan Chen, Marc R Gartenberg

**Affiliations:** Department of Biochemistry and Molecular Biology, Robert Wood Johnson Medical School, Rutgers University, Piscataway, United States; Department of Biochemistry and Molecular Biology, Robert Wood Johnson Medical School, Rutgers University, Piscataway, United States and The Cancer Institute of New Jersey, New Brunswick, United Statesmarc.gartenberg@rutgers.edu

**Keywords:** epigenetics, bistability, Sir2, Sir3, Sir4, histone H3, *S. cerevisiae*

## Abstract

Silent chromatin in budding yeast is propagated from one generation to the next, even though ‘silenced’ genes are occasionally expressed.

**Related research article** Dodson AE, Rine J. 2015. Heritable capture of heterochromatin dynamics in *Saccharomyces cerevisiae*. *eLife* **4**:e05007. doi: 10.7554/eLife.05007

**Image** A new assay employs a permanent switch from red to green fluorescence to mark yeast cells that ‘derepress’ silenced genes

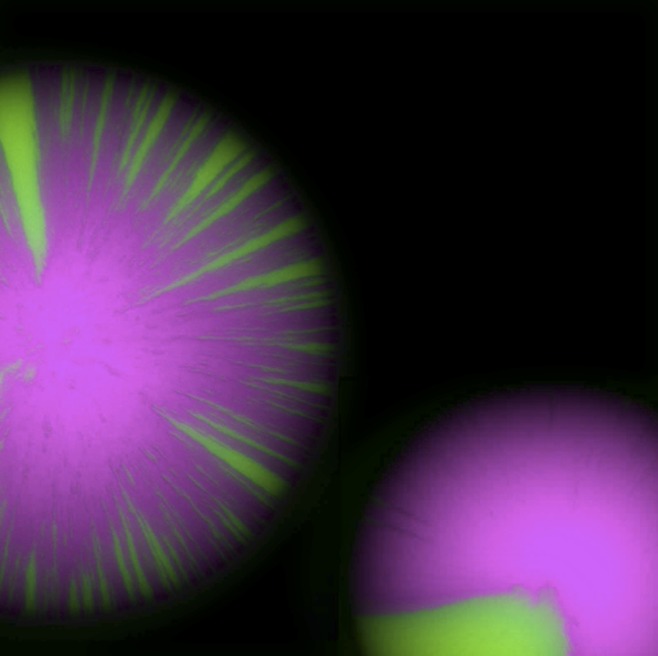

If a tree falls in a forest and no one is there to hear it, does it make a sound? While questions about the perception and generation of sound can be debated endlessly, one thing is certain: a tree did fall. If a tree only swayed in a forest, how would we know? Sometimes events transpire that leave no evidence of their occurrence.

DNA in eukaryotic cells is wrapped around histone proteins to form a structure called chromatin. Heterochromatin is a more condensed form of chromatin that silences the genes encoded in the DNA, and is inherited by new cells via a code of chemical marks on the histones and sometimes the DNA. Female mammals convert one of their two X chromosomes into heterochromatin, which stops them from expressing a double dose of all X-linked genes for a lifetime. Likewise, a heterochromatin-like structure in budding yeast, termed ‘silent chromatin’, controls the yeast's sexual identity via faithful gene silencing. Now, in *eLife*, Anne Dodson and Jasper Rine—from the University of California, Berkeley—report that budding yeast occasionally breaks this wall of silence (Dodson and Rine, 2015).

During mating of the budding yeast, *Saccharomyces cerevisiae*, cells of opposite mating-types fuse with one another. A yeast cell's mating type can be *MATα* or *MAT***a**, depending on which pair of genes is at the *MAT* locus in its genome (Figure 1A). Identical, but silenced, copies of the mating-type genes are found at two other genome locations. Budding yeast use these ‘hidden *MAT* loci’ as templates to replace the genes at the *MAT* locus when switching between mating types. The hidden *MAT* loci have been studied for decades to understand how chromatin shuts off genes.

**Figure 1.**
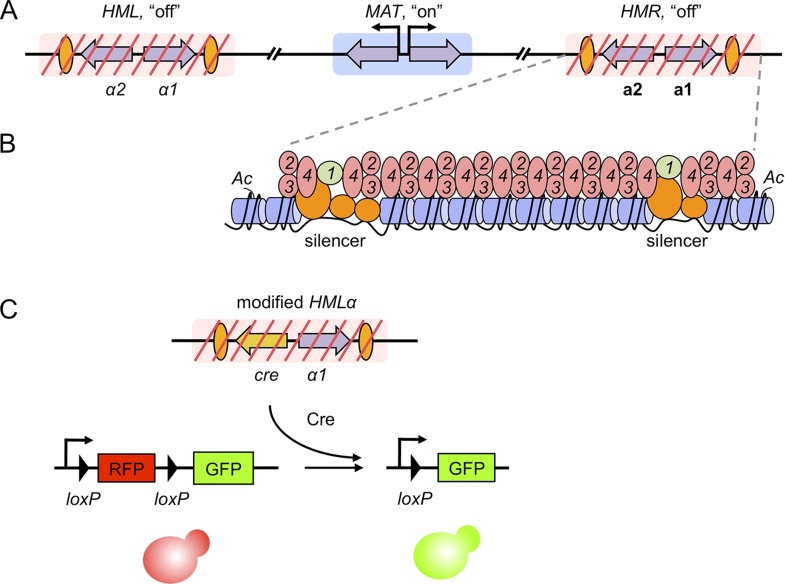
Gene silencing in budding yeast, and the recombinase assay used to record transient derepression. (**A**) The mating-type loci of budding yeast. The two silenced hidden *MAT* loci, *HML* and *HMR*, are indicated by red slashes and contain the *α* and **a** mating-type genes (purple arrows), respectively. The expressed *MAT* locus can contain either set of genes. Orange ovals depict sections of DNA called silencers. (**B**) The distribution of silencing proteins at one hidden *MAT* locus *HMR*. Silencer binding proteins (orange) together with Sir1 (light green) recruit the Sir complex, composed of Sir2, Sir3 and Sir4 (pink). The complex spreads out from silencers; binding to and de-acetylating histone proteins (blue cylinders) in the chromatin, which results in gene silencing. (**C**) Dodson and Rine devised a new assay to measure transient derepression of a silenced locus. They replaced *α2* in *HML* with the coding sequence of an enzyme called Cre recombinase (yellow arrow). If *HML* derepresses, even briefly, the recombinase is expressed. The Cre enzyme recognizes two *loxP* sequences (arrowheads) in a reporter construct elsewhere in the genome, causing a rearrangement in the construct that removes RFP and activates GFP. This means that transient derepression events are recorded by a permanent switch from red to green fluorescence.

Nearby sections of DNA, termed silencers, distinguish the hidden *MAT* loci from all other locations in the yeast genome (Rusché et al., 2003). Proteins bind to the silencers, and recruit a set of proteins called the Sirs. Sir2, Sir3 and Sir4 proteins form a large complex that spreads out from the silencers by binding to, and modifying, the surrounding histones (Figure 1B). An additional Sir protein, called Sir1, remains at the silencers to help recruit the other Sirs. Gene silencing by Sir proteins reduces the expression of ‘silenced’ genes below detectable limits and renders the DNA almost entirely inaccessible. Typically, non-silenced cells are found about as often as the spontaneous mutations that inactivate the silencing process.

To the casual observer, silent chromatin might thus seem inert and unchanging. Two previous observations, however, suggested that this is not the case. First, silencing proteins constantly move onto, and off of, heterochromatin (Cheng and Gartenberg, 2000; Cheutin et al., 2003; Festenstein et al., 2003). Second, reporter genes placed within silent chromatin can be artificially prodded toward expression during certain stages of the cell cycle (Aparicio and Gottschling, 1994)—suggesting that heterochromatin might occasionally break its silence.

Do the dynamic properties of silent chromatin ever permit silenced mating-type genes to be expressed? To address the question, Dodson and Rine developed an elegant assay that means even a rare event—a momentary loss of silencing in this case—is marked in a way that persists and is easily detectable. They introduced the coding sequence of an enzyme called Cre recombinase into the hidden *MAT* loci (Figure 1C). On a different chromosome, Dodson and Rine assembled a reporter construct from the genes for red and green fluorescent proteins (i.e. RFP and GFP) that works as follows: if silencing is lost in a cell, the recombinase removes the RFP gene and causes that cell, and all its descendants, to permanently switch from red to green fluorescence. Recombinases have been used similarly in the past to detect gene expression in bacterial pathogens during infection (Camilli et al., 1994).

This assay revealed that ‘silenced’ yeast genes are expressed, or ‘derepressed’, in wild-type cells, as Dodson and Rine observed green fluorescent sectors within otherwise red fluorescent yeast colonies. Each green sector marks when silencing was briefly lost, and looking at how often these sectors appeared suggests that such events are rare, occurring roughly once out of every 1000 cell divisions. The assay was used to reveal that Hst3, an enzyme closely related to Sir2 that also modifies histones, also contributes to silencing of the hidden *MAT* loci. Mutants without the gene for Hst3 develop seven times as many green sectors as wild-type yeast, but fail to register derepression in conventional assays (Yang et al., 2008). This illustrates the sensitivity of the recombinase approach.

Dodson and Rine also looked at how often they could detect the expression of Cre recombinase within individual cells using a technique called ‘single-molecule RNA FISH’ (Raj et al., 2008). In most cells, they detected nothing more than background. However, in about one in every 2500 cells (about as often sectors formed in their colony assay), they found Cre recombinase was expressed at roughly 25% of the level reached in cells that cannot silence at all. The data indicate that rare, sub-maximal bursts of transcription occur within chromatin that has been silenced.

Silencing is viewed as the culmination of three steps: a silent state is first established, then maintained and finally passed on to the cell's descendants. Sir2, Sir3 and Sir4 participate in all three steps, but Sir1 is different. In mutant yeast without Sir1, silencing is lost and not restored efficiently in some cells, whereas in other cells silencing is propagated from generation to generation. This suggests that silencing cannot be established well without Sir1 (Pillus and Rine, 1989; Xu et al., 2006). Dodson and Rine revisited the role of Sir1 using their sensitive sector-based assay and found that silencing cannot be properly maintained and/or inherited without Sir1 either. But how might this work? It's possible that Sir1 counteracts common disruptions to silent chromatin, like DNA replication, and rapidly re-establishes the silent state each cell cycle. Alternatively, Sir1 might continuously replace any silencing proteins that are normally lost from heterochromatin. In this regard, it is significant that silencers, where Sir1 binds, also continuously maintain the silent state, even in the absence of DNA replication (Cheng and Gartenberg, 2000).

Evolution has not selected for a more durable silent chromatin, most likely because of a need to balance silencing with other cellular processes. Silent chromatin must be flexible enough to permit the passage of the DNA replication machinery and allow mating-type switches. Moreover, if the Sir protein complex could associate with histones tightly enough to block all gene expression, then non-specific binding by the complex might silence the wrong genes elsewhere in the genome. Instead, evolution settled upon a heterochromatin-like structure at the hidden *MAT* loci that is dynamic and permits breaks in the silence that had never been heard before now.

## References

[bib1] Aparicio OM, Gottschling DE (1994). Overcoming telomeric silencing: a trans-activator competes to establish gene expression in a cell-cycle dependent way. Genes & Development.

[bib2] Camilli A, Beattie DT, Mekalanos JJ (1994). Use of genetic recombination as a reporter of gene expression. Proceedings of the National Academy of Sciences of USA.

[bib3] Cheng TH, Gartenberg MR (2000). Yeast heterochromatin is a dynamic structure that requires silencers continuously. Genes & Development.

[bib4] Cheutin T, McNairn AJ, Jenuwein T, Gilbert DM, Singh PB, Misteli T (2003). Maintenance of stable heterochromatin domains by dynamic HP1 binding. Science.

[bib5] Dodson AE, Rine J (2015). Heritable capture of heterochromatin dynamics in *Saccharomyces cerevisiae*. eLife.

[bib6] Festenstein R, Pagakis SN, Hiragami K, Lyon D, Verreault A, Sekkali B, Kioussis D (2003). Modulation of heterochromatin protein 1 dynamics in primary mammalian cells. Science.

[bib7] Pillus L, Rine J (1989). Epigenetic inheritence of transcriptional states in *Saccharomyces cerevisiae*. Cell.

[bib8] Raj A, van den Bogaard P, Rifkin SA, van Oudenaarden A, Tyagi S (2008). Imaging individual mRNA molecules using multiple singly labeled probes. Nature Methods.

[bib9] Rusché LN, Kirchmaier AL, Rine J (2003). The establishment, inheritance, and function of silenced chromatin in *Saccharomyces cerevisiae*. Annual Review of Biochemistry.

[bib10] Xu EY, Zawadzki KA, Broach JR (2006). Single-cell observations reveal intermediate transcriptional silencing states. Molecular Cell.

[bib11] Yang B, Miller A, Kirchmaier AL (2008). *HST3*/*HST4*-dependent deacetylation of lysine 56 of histone H3 in silent chromatin. Molecular Biology of the Cell.

